# Supplementary material for: Establishing and validating syndromic surveillance of gastrointestinal infections using routine emergency department data, Germany, 2019–2023
Source: Sci Rep. 2025 Nov 3;15:38281. doi: 10.1038/s41598-025-13675-z (PMC12583625; doi:10.1038/s41598-025-13675-z)
Supplement: Supplementary file 1 — Supplementary Information. [file 41598_2025_13675_MOESM1_ESM.docx]

Supplementary Table 1: Frequency of ICD-10 codes in visits (n = 10,352) selected based on presenting complaint “254 – Diarrhea”. ICD-10 codes with frequencies ≤1% were not shown. Frequencies were calculated relative to all visits with available ICD-10 codes. Germany, 2019 – 2023.

| ICD-10 codes | | *n* | Frequency % |
| --- | --- | --- | --- |
| **A09.9** | Other and unspecified gastroenteritis and colitis of unspecified origin | 3,093 | 29.9 |
| **E86** | Volume depletion | 504 | 4.9 |
| **A09.0** | Other and unspecified gastroenteritis and colitis of infectious origin | 503 | 4.9 |
| **R11** | Nausea and vomiting | 334 | 3.2 |
| **Z29.0** | Isolation as prophylactic measure | 310 | 3.0 |
| **Z11** | Special screening examination for infectious and parasitic diseases | 206 | 2.0 |
| **R10.4** | Other and unspecified abdominal pain | 187 | 1.8 |
| **R53** | Malaise and fatigue | 185 | 1.8 |
| **E87.1** | Hypo-osmolality and hyponatraemia | 127 | 1.2 |
| **E87.6** | Hypokalaemia | 125 | 1.2 |
| **N17.9** | Acute renal failure, unspecified | 111 | 1.1 |
| **N39.0** | Urinary tract infection, unspecified | 107 | 1.0 |
| **K52.9** | Noninfective gastroenteritis and colitis, unspecified | 106 | 1.0 |

Supplementary Table 2: Frequency of ICD-10 codes for visits (n = 10,696) selected based on presenting complaint “257 – Nausea and/or vomiting”. ICD-10 codes with frequencies ≤1% were not shown. Frequencies were calculated relative to all visits with available ICD-10 codes. Germany, 2019 – 2023.

| ICD-10 codes | | *n* | Frequency % |
| --- | --- | --- | --- |
| **R11** | Nausea and vomiting | 2,194 | 20.5 |
| **A09.9** | Other and unspecified gastroenteritis and colitis of unspecified origin | 867 | 8.1 |
| **R10.4** | Other and unspecified abdominal pain | 308 | 2.9 |
| **R53** | Malaise and fatigue | 229 | 2.1 |
| **E86** | Volume depletion | 224 | 2.1 |
| **R10.1** | Pain localized to upper abdomen | 192 | 1.8 |
| **A09.0** | Other and unspecified gastroenteritis and colitis of infectious origin | 179 | 1.7 |
| **R42** | Dizziness and giddiness | 152 | 1.4 |
| **K92.0** | Haematemesis | 144 | 1.3 |
| **Z11** | Special screening examination for infectious and parasitic diseases | 141 | 1.3 |
| **N39.0** | Urinary tract infection, unspecified | 128 | 1.2 |
| **E87.1** | Hypo-osmolality and hyponatraemia | 122 | 1.1 |


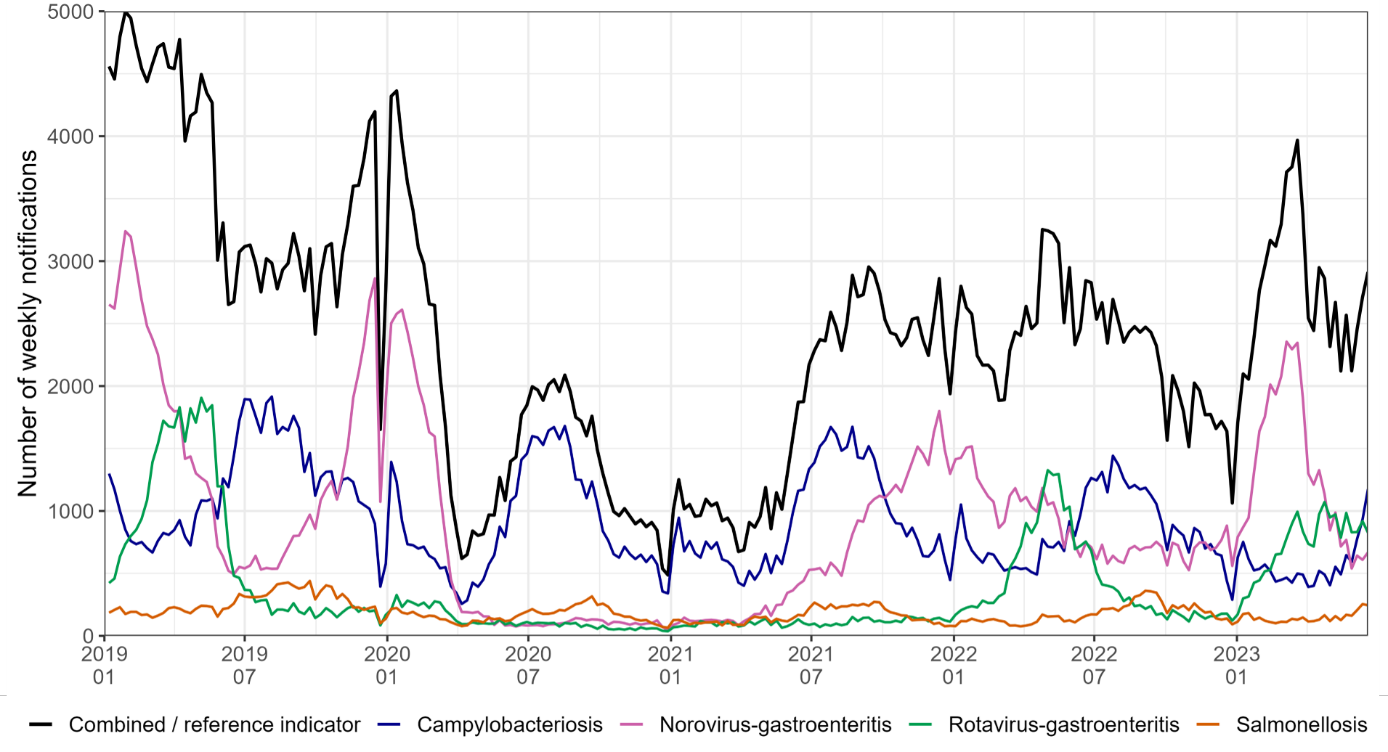
Supplementary Figure 1: Notifications for norovirus-gastroenteritis, rotavirus-gastroenteritis, salmonellosis and campylobacteriosis from laboratory-based surveillance and derived combined reference indicator. Germany, between week 01/2019 and up to week 23/2023.


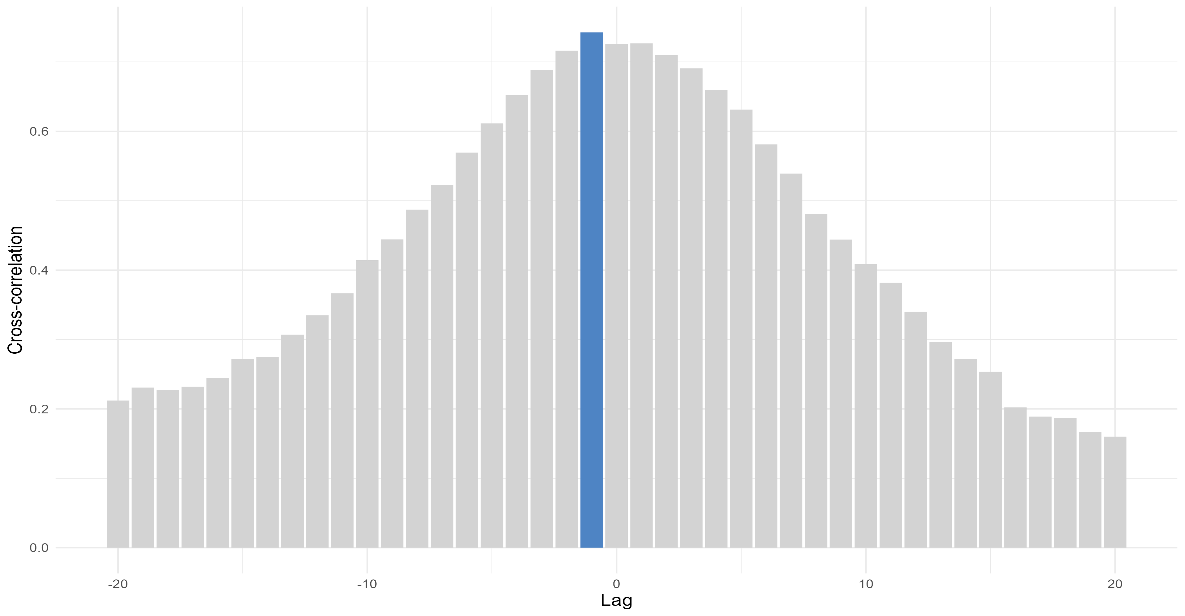


Supplementary Figure 2: Cross-correlation analysis between the emergency department indicator trend and the combined reference indicator trend, by lag (in weeks). The (highest) cross-correlation at lag -1 is highlighted in blue. Germany, 2019 – 2023.
